# Transcriptome Analysis of Flowering Time Genes under Drought Stress in Maize Leaves

**DOI:** 10.3389/fpls.2017.00267

**Published:** 2017-03-01

**Authors:** Kitae Song, Hyo Chul Kim, Seungho Shin, Kyung-Hee Kim, Jun-Cheol Moon, Jae Yoon Kim, Byung-Moo Lee

**Affiliations:** ^1^Department of Life Science, Dongguk University-SeoulSeoul, South Korea; ^2^Agriculture and Life Sciences Research Institute, Kangwon National UniversityChuncheon, South Korea; ^3^Department of Plant Resources, College of Industrial Science, Kongju National UniversityYesan, South Korea

**Keywords:** maize, flowering time, drought, RNA-seq, alternative splicing

## Abstract

Flowering time is an important factor determining yield and seed quality in maize. A change in flowering time is a strategy used to survive abiotic stresses. Among abiotic stresses, drought can increase anthesis-silking intervals (ASI), resulting in negative effects on maize yield. We have analyzed the correlation between flowering time and drought stress using RNA-seq and bioinformatics tools. Our results identified a total of 619 genes and 126 transcripts whose expression was altered by drought stress in the maize B73 leaves under short-day condition. Among drought responsive genes, we also identified 20 genes involved in flowering times. Gene Ontology (GO) enrichment analysis was used to predict the functions of the drought-responsive genes and transcripts. GO categories related to flowering time included reproduction, flower development, pollen–pistil interaction, and post-embryonic development. Transcript levels of several genes that have previously been shown to affect flowering time, such as *PRR37*, transcription factor *HY5*, and *CONSTANS*, were significantly altered by drought conditions. Furthermore, we also identified several drought-responsive transcripts containing C_2_H_2_ zinc finger, CCCH, and NAC domains, which are frequently involved in transcriptional regulation and may thus have potential to alter gene expression programs to change maize flowering time. Overall, our results provide a genome-wide analysis of differentially expressed genes (DEGs), novel transcripts, and isoform variants expressed during the reproductive stage of maize plants subjected to drought stress and short-day condition. Further characterization of the drought-responsive transcripts identified in this study has the potential to advance our understanding of the mechanisms that regulate flowering time under drought stress.

## Introduction

Maize (*Zea mays* L.) is a major crop used worldwide as a source of food, fuel, and animal feed. Maize serves as a key resource for economic and industrial applications, in addition to its role as food. Flowering time is an important feature of maize production that is widely known to affect productivity and seed quality. With respect to maize reproduction, 1 week before silking until 2 weeks after silking represents a key period, during which abortion of ovules and the fertilization rate are determined, and kernels and ears occur. Pathways required for flowering have response systems that mediate survival under various stresses. In response to stress, such as drought, flowering pathways are accelerated to produce flowers and seeds more rapidly (Franks et al., 2007; Bernal et al., 2011; Franks, 2011; Krasensky and Jonak, 2012). Drought stress has many negative effects, such as decreasing carbon availability flower drop, pollen death, ovule abortion, and the anthesis-silking interval (ASI) during the reproductive stages (Hall et al., 1984; Blum, 1996; Andrade et al., 1999). Drought-induced increases in the ASI negatively affect the fertilization rate, kernel filling, and seed quality and weight (Byrne et al., 1995; Bolaños and Edmeades, 1996; Edmeades et al., 1997; Bruce et al., 2002). For this reason, ASI is frequently used as an index of drought tolerance.

To improve production under drought stress, several attempts to improve varieties have been undertaken using recombinant inbred lines (RIL), molecular markers, and quantitative trait locus (QTL) mapping (Cattivelli et al., 2008; Yu et al., 2008; McMullen et al., 2009). However, the complexity of the maize genome and the lack of knowledge of the specific genetic mechanisms underlying drought tolerance remain a major challenge. Consistent with the diversity of these highly heterozygous plants and their genomic complexity, different genotypes have been found to exhibit different response systems under drought stress (Shinozaki and Yamaguchi-Shinozaki, 2007; Hansey et al., 2012; Jiang et al., 2012). Therefore, to improve our understanding of the relationship between drought and yield, our goal was to characterize the mechanisms associated with responses to drought that impact flowering time.

The flowering time determining the ASI of maize is closely related to flowering time genes. Genes such as FLOWERING LOCUS C (FLC), CONSTANS (CO), GIGANTEA (GI), and SUPPRESSOR of OVEREXPRESSION of CONSTANS1 (SOC1) were known to regulate flowering time (Kim et al., 2006; Buckler et al., 2009; Jung and Müller, 2009; Kong et al., 2010; Endo-Higashi and Izawa, 2011; Hung et al., 2012; Ito et al., 2012; Li et al., 2016). Expression of these genes controlled by photoperiod, light sensing, circadian, and various stress (Johnson et al., 1994b; Datta, 2006; Fornara et al., 2009; Jung and Müller, 2009; Meng et al., 2011; Hung et al., 2012; Coelho et al., 2013; Chao et al., 2014). Similarly, drought known make changes of ASI, therefore drought maybe affects the flowering time genes. Changes of the expression level of the flowering time genes due to drought have been observed (Franks et al., 2007; Zhuang et al., 2007; Kakumanu et al., 2012; Corrales et al., 2014; Kooyers, 2015). A more detailed analysis of gene expression pattern can be lead to improving our understanding of the regulation of processes associated with flowering time under drought condition.

Gene expression studies that characterize the response to drought stress in maize have been attempted in various tissues (Zheng et al., 2004; Poroyko et al., 2007; Zhuang et al., 2007) and during multiple stages (Zheng et al., 2004; Yue et al., 2008; Humbert et al., 2013). With the advent of RNA sequencing (RNA-seq) technologies and publication of the reference genome (Schnable et al., 2009), we now have access to a great deal of information regarding the diversity of the genome and gene expression in maize, including structural variations, differing response systems, and alternative splicing events (Wang et al., 2009; Jain, 2011). Gene annotations and sequences included in the reference genome have made improved genomic studies possible. Recently, RNA-seq has been used to characterize transcriptional changes resulting from abiotic stress (Krasensky and Jonak, 2012; Frey et al., 2015; Liu et al., 2015), and the differentially expressed genes (DEGs) identified were found to be useful for predicting differences in tolerance between maize cultivars. Alternative splicing (AS) also plays an important role in stress responses, and AS events have been estimated to occur for more than 60% of intron-containing genes in *Arabidopsis thaliana* (Filichkin et al., 2010). AS is also known to occur in a differential manner in distinct tissues, developmental stages, and stress responses in plants (Li et al., 2013; Staiger and Brown, 2013; Garg et al., 2016; Shankar et al., 2016).

In this study, we used RNA-seq to identify transcriptional variations between maize plants subjected to well-watered (WW) and drought-stressed (DS) conditions under short-day condition. The resulting RNA-seq data was analyzed using bioinformatics tools to identify changes in gene expression and alternative splicing under drought stress. Differentially-expressed genes were subjected to BLAST analysis to predict their functions. We then focused on drought-responsive genes known or predicted to be involved in flowering time and analyzed the potential of these genes to change flowering time in response to drought stress.

## Materials and methods

### Plant growth and drought conditions

Maize (*Zea mays* cv. B73) plants were grown in 10-L pots (37 × 37.5 × 22 cm). The plants were maintained in a greenhouse and grown under short-day condition until top of the tassel was visible. The short-day condition was set up for 12 h light and 12 h dark and temperate varied from 26 to 28°C during day and 23–25°C during night. When the tassel was visible, the plants were divided into two groups: the well-watered (WW) and drought-stressed (DS) groups. The WW pots were maintained at potentials >−0.2 MPa (15–20% soil water content) with irrigation, while DS pots were maintained at <−1.5 MPa (5–8% soil water content). When pollen shed began, leaf tissue was harvested from top collared leaf (flag leaf) from three different plants in each replicate group and condition. As a result, drought caused changes of ASI to be 3–4 days and the samples from each condition harvested on different days. On average, DS group was underlying more than 15 days under drought condition. All leaves were frozen in liquid nitrogen immediately after collecting and were then transferred to a deep freezer to be stored at −80°C.

### RNA isolation and RNA-seq

To construct RNA libraries, leaves collected from WW, and DS plants were subjected to RNA isolation with biological replication. Total RNA was prepared from each leaf using the RNeasy Plant Mini Kit (Qiagen). RNA quality and integrity was assessed using a 2100 Bioanalyzer RNA Nanochip (Agilent Technologies) prior to RNA-seq. RNA-seq was then performed using the Illumina HiSeq platform.

### Bioinformatics

All reads from each sample were aligned to the maize reference genome [B73_RefGen_v3 annotation build (5b+)] using default parameters in Tophat2. The Tophat methodology facilitates the identification of splicing events. Aligned sequences from Tophat2 (Kim et al., 2013) were assembled separately using Cufflinks 2.2.1 (Trapnell et al., 2012). Cufflinks assembles specific isoforms based on alternative splicing events. Results from Cufflinks were compared with annotation of the reference genome (http://plants.ensembl.org/Zea_mays/) using Cuffmerge. Class codes obtained from Cuffmerge outputs were used to identify novel isoforms resulting from newly identified splice junctions and intergenic transcripts.

Differentially expressed genes (DEGs) were identified by calculating fragments per kilobase of transcript per million mapped reads (FPKM) values. DEGs were defined as genes having a false discovery rate (FDR) (Benjamini and Yekutieli, 2001) <0.001 and an absolute log_2_ fold change value >1. To further characterize genes identified as being differentially expressed in response to drought stress, DEGs and consensus sequences of isoform were mapped to GO classifications using Blast2GO (Conesa and Götz, 2008). Three categories of GO annotations were analyzed: biological process, molecular function, and cellular component. GO enrichment analysis was performed with BiNGO plugins for Cytoscape (Maere et al., 2005) using the hypergeometric test and the Bonferrony correction method. Bonferrony correction was employed for *P*-value correction with a cut-off of 0.05. To identify alternative splicing events associated with drought stress, Cuffdiff data was analyzed using the spliceR module in R (Vitting-Seerup et al., 2014).

### Quantitative real-time polymerase chain reaction (qRT-PCR) validation of RNA-seq data

RNA isolated from the WW and DS groups was used to construct a cDNA library. First-strand synthesis was performed using the PrimeScript First Strand cDNA Synthesis kit (Takara) and random primers according to the manufacturer's recommended procedures. qRT-PCR was carried out using the CFX Connect™ Real-Time PCR Detection System (Bio-Rad). The CT-method was used to quantify changes in gene expression, and the maize 18s rRNA and Actin1 served as references for normalization. Expression was analyzed for each of the selected genes under both conditions (WW and DS). Three independent biological replicates of each sample were subjected to qRT-PCR analysis. To determine relative fold changes, gene expression was normalized to CT-values for the reference genes. The CT-values for each gene under each condition were calculated using ΔΔCT method as previously described (Livak and Schmittgen, 2001).

## Results

### RNA-seq analysis reveals changes in the maize transcriptome in response to drought stress

RNA samples from well-watered (WW) and drought-stressed (DS) plants were subjected to RNA-seq using an Illumina Hi-Seq 2000 instrument. A total of six samples were analyzed, which included three biological replicates for each condition. For each replicate, 99–113 million reads were obtained. More than 280 million paired reads were utilized for comparative analysis, with approximately 47 million reads from each sample (Table 1). Of these reads, 86.7–89.0% could be mapped to the reference genome (*Zea mays* AGPv3.30) as unique and multiple matches. Mapped reads were analyzed using the Cufflinks pipeline (Trapnell et al., 2012) to reveal DEGs, alternative splicing events, and novel transcripts. Expression values associated with genes and transcripts were calculated using the fragments per kilobase of exon per million fragments mapped (FPKM) model. Also expression of *ZmDREB2A* was used to validate for the plants applied drought stress (Figure S1).

**Table 1:** **Summary of RNA-seq data from WW and DS samples**.

**Cultivar**	**Sample ID**	**Total read bases (bp)**	**Total reads (single reads)**	**Aligned pairs**	**Multiple alignments**	**Discordant**	**Rate of concordant pairing**
B73	WW-1	10,246,560,090	101,451,090	45,700,180	2,121,199	673,769	88.8
	WW-2	10,249,098,422	101,476,222	46,020,143	2,484,676	878,517	89.0
	WW-3	11,496,381,156	113,825,556	51,076,080	2,366,476	691,398	88.5
	DS-1	11,227,197,572	111,160,372	50,012,836	2,201,247	675,166	88.8
	DS-2	10,035,278,190	99,359,190	44,618,507	2,156,617	954,509	87.9
	DS-3	10,600,680,230	104,957,230	47,021,361	3,850,820	1,521,877	86.7

### Transcriptome analysis of drought-responsive genes

**Figure 1:**
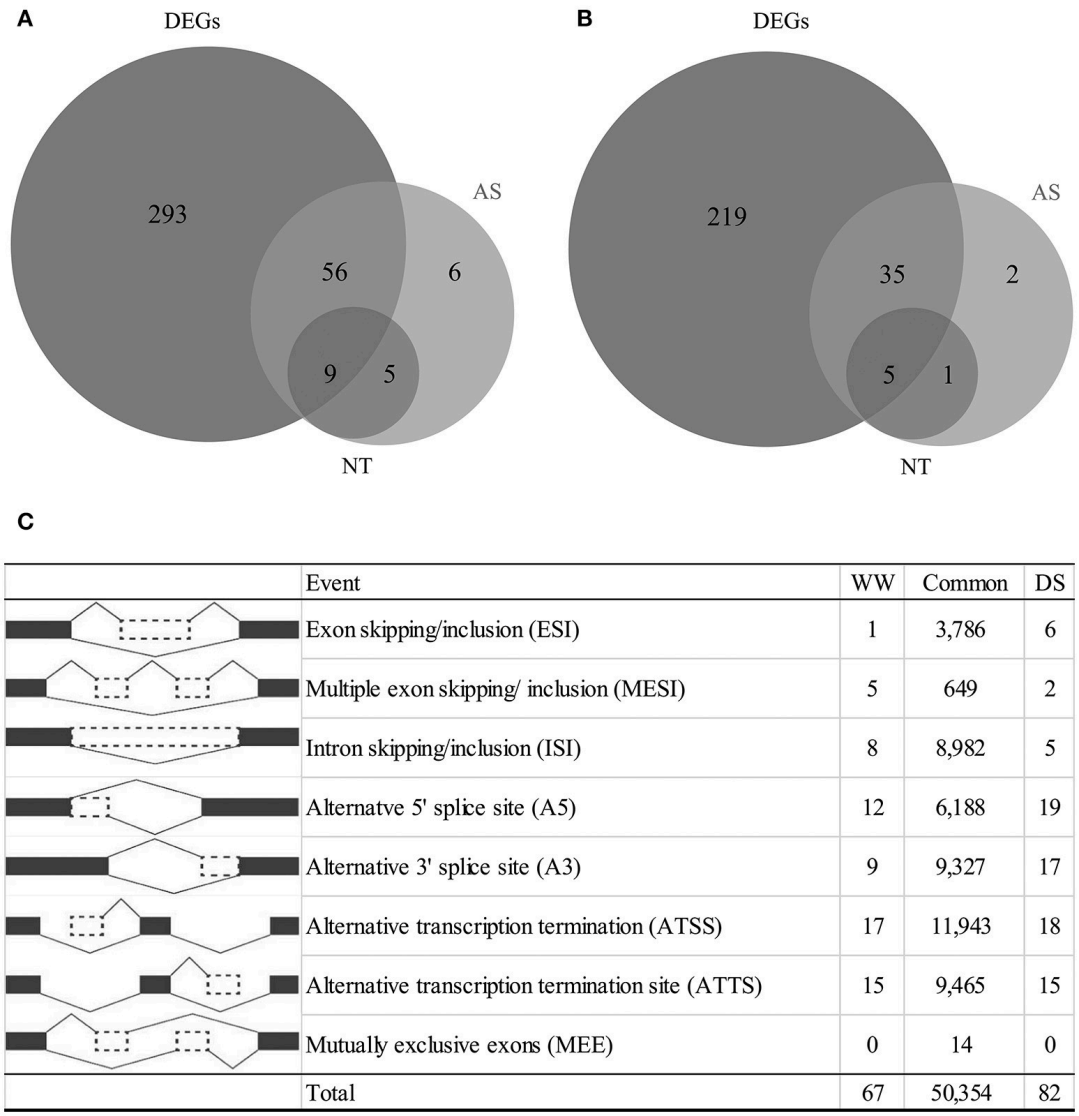
**Summary of differentially expressed genes, alternative splicing events and novel transcripts (NT) under drought condition**. **(A)** Number of genes as up regulation, **(B)** number of genes as down regulation, **(C)** number of alternative splicing events classified by each type.

To characterize transcriptional variations that occur in response to drought stress, we conducted differential gene expression analysis. A total of 34,002 genes were expressed under both WW and DS conditions. Of these genes, 954 passed the Cuffdiff test and were considered to be drought-responsive genes. DEGs, which exhibited a log_2_ fold change >1, included 617 genes (Figure 1), 358 of which were upregulated, and 259 of which were downregulated (Figure 1, Table S1). Of the DEGs, 512 could be matched to the reference genome, and 105 could not.

A total of 92,438 alternatively-spliced transcripts were identified. SpliceR was used to identify significantly changed isoforms. Filtering resulted in the identification of 50,354 shared AS events, 67 WW-specific AS events, and 82 DS-specific AS events (Figure 1C). Alternative transcription termination sites (ATTS) and alternative transcription start sites (ATSS) were the most predominant AS events, making up 11,943 and 9,465 events, respectively (Figure 1C). Other AS events identified included 9,327 alternative 3′ splice site events (A3), 6,185 alternative 5′ splice site events (A5), 8,982 intron skipping/retention events (ISI), and 3,786 exon skipping/inclusion events (ESI). The types of AS events varied by condition, with the A3, A5, and ESI types being more prevalent under drought conditions. A total of 177 drought-responsive isoforms were identified as having *q* < 0.05. To characterize whether alternatively spliced transcripts were significantly differentially expressed under drought conditions, we selected 126 transcripts with log_2_ fold changes >1 (Table S2). Of these AS transcripts, 82 (from 76 genes) were upregulated, and 44 (from 43 genes) were downregulated (Figure 1). Generally, genes with AS isoforms were also identified as DEGs; however, 14 genes were found only to be differentially spliced. Most genes had a single differentially expressed isoform, but six upregulated genes and one downregulated gene had two differentially expressed isoforms. Some genes were found to have novel transcripts that were differentially regulated under drought conditions. Of the upregulated transcripts, 14 were novel transcripts, including nine transcripts associated with DEGs (Figure 1A). In contrast, six novel transcripts were downregulated (Figure 1B).

### GO enrichment of differentially expressed genes and transcripts

**Figure 2:**
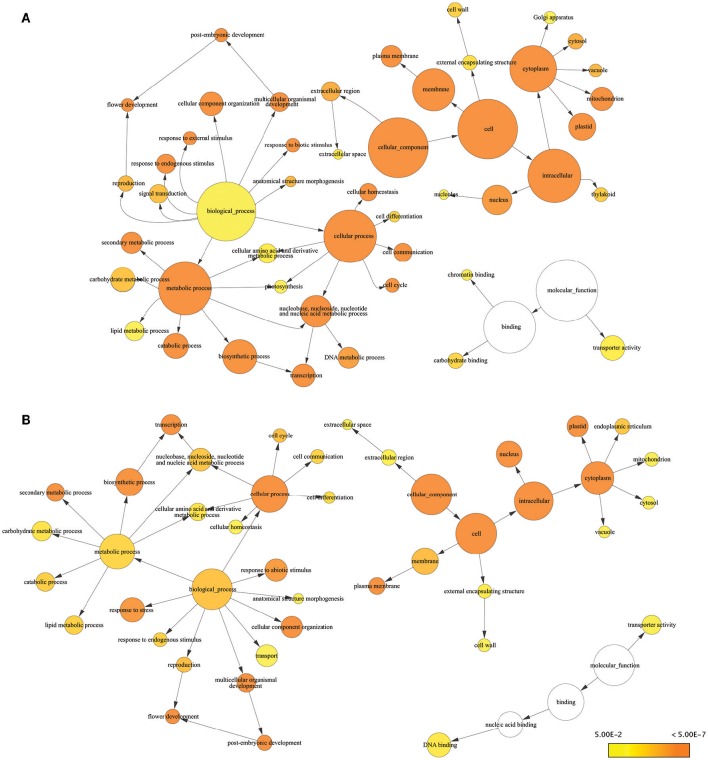
**Enrichment analysis of responsive genes and transcripts using GO terms from GO Slim**. Significantly overrepresented GO Slim terms were visualized by BiNGO as app in Cytoscape. The size of a node was proportional to the number of targets in GO category. The color of the node represents the significance of enrichment: higher significance was represented with deeper color. **(A)** Result of DEGs **(B)** result of responsive transcripts.

Consensus sequences for drought-responsive genes and transcripts obtained from RNA-seq results were subjected to Blast2Go for further analysis of gene function. Many of the DEGs we identified were known stress-responsive genes. Almost all of the drought-responsive genes we identified were expressed at levels similar to previous reports characterizing gene expression in response to drought stress using genome-wide association and QTL mapping (Shinozaki and Yamaguchi-Shinozaki, 2007; Yue et al., 2008; Setter et al., 2010; Kakumanu et al., 2012; Thirunavukkarasu et al., 2014). GO terms associated with biological process for DEGs included response to stress, response to endogenous stimulus, photosynthesis, chromatic binding, and response to external stimulus, which are consistent with drought stress (Figure 2A). GO terms related to reproductive stages included flower development (six genes), reproduction (four genes), pollen–pistil interaction (one gene), and post-embryonic development (one gene) (Table S3). GO terms from the molecular function category involved in response to stress included carbohydrate binding, transporter activity, and chromatin binding.

Known drought-responsive genes found in our results included the genes encoding alpha- and beta-amylase (GRMZM2G138464 and GRMZM2G450125), which break down starch, a process that has been shown to be upregulated under drought stress (Krasensky and Jonak, 2012; Prasch et al., 2015). Two genes involved in the ABA biosynthetic pathway (GRMZM5G858784 and GRMZM2G179147) exhibited increased transcript levels under drought conditions (Yue et al., 2008; Urano et al., 2009). We also found that expression of the gene encoding sucrose synthase 7 (GRMZM2G060583) was downregulated under drought conditions (Table S1). In a previous study, genes encoding sucrose synthase were downregulated in ovary tissue, but exhibited no drought-mediated changes in the leaf meristem (Kakumanu et al., 2012).

In addition to DEGs, we found 126 transcripts with significantly different expression in response to drought. Like the DEGs, transcripts known to be involved in the drought response exhibited altered regulation under drought conditions. For example, beta-amylase (GRMZM2G450125), chitinase (GRMZM2G005633), carotenoid hydroxylase (GRMZM2G164318), WRKY transcripts (GRMZM2G120320 and GRMZM2G018487), and heat shock proteins (GRMZM2G024668 and GRMZM2G428391) were upregulated by drought. These proteins have been reported to play important roles in the response to drought stress (Hu et al., 2009; Banerjee and Roychoudhury, 2015; Prasch et al., 2015). Differentially expressed isoforms were associated with many of the same GO terms as DEGs. Biological process GO terms associated with differentially expressed isoforms included response to stress, response to abiotic stimulus, and response to endogenous stimulus. We also identified several novel stress-induced transcript isoforms. Of the 14 novel isoforms that exhibited significantly different expression, isoform of six transcripts (GRMZM2G162598_T01, GRMZM2G045239_T01, GRMZM2G163809_T02, GRMZM5G846082_T01, GRMZM2G068519_T01, and GRMZM2G099305_T01) had no annotations based on BLAST results, five upregulated novel isoforms (GRMZM2G025322_T01, GRMZM2G140355_T01, GRMZM2G154580_T01, AC233865.1_FGT001, and GRMZM2G103250_T01) were associated with response to stress as a biological process, and four upregulated isoforms (GRMZM2G140355_T01, GRMZM2G061419_T01, AC233865.1_FGT001, and GRMZM2G106945_T01) were associated with DNA and protein binding according to molecular function (Table S2). Several transcript-associated GO terms were identified that were related to specific reproductive stages, including pollination, flower development, and post-embryonic development in the biological processes category (Table S4). A total of 13 transcripts were found to have GO terms related to reproductive stages. Twelve of these genes were also identified as DEGs. The exception was GRMZM2G140355, which only exhibited isoform-specific changes.

### Transcriptome changes in drought-responsive genes associated with flowering time

Genes that play a role in regulation of flowering time have been reported in various studies. Recently, a total of 919 candidate genes related to maize flowering time were reported by Li et al. based on analysis of published data (Li et al., 2016). The candidate genes were contains genes such as ZmCCT and ZCN8 are well-known flowering time regulators in maize (Meng et al., 2011; Hung et al., 2012), and homologs of many genes that have been shown to regulate flowering time in other plants, such as CONSTANS (CO), FLOWERING LOCUS T (FT), MADS-box, and EARLY FLOWERING (Dong et al., 2012; Hung et al., 2012). To assess changes in these previously reported flowering time genes under drought conditions, we compared expression data for these genes from WW reproductive stage plants and those under DS (Table S4). A total of 792 genes are expressed in both conditions, and 172 genes were not expressed. Among the 792 genes, only 19 genes exhibited significant changes under drought conditions. Thirteen genes (GRMZM2G047055, GRMZM2G154580, GRMZM2G062458, AC208915.3_FG010, GRMZM2G104549, GRMZM2G176173, GRMZM2G137046, GRMZM2G389155, GRMZM2G069146, GRMZM2G005459, GRMZM2G134941, GRMZM2G004483, and GRMZM2G092363) were upregulated, and six genes (GRMZM2G414192, GRMZM2G021777, GRMZM2G012717, GRMZM2G161680, GRMZM2G107886, and GRMZM2G142718) were downregulated (Table S1). We can also find homologs of the genes from model organisms (Table S5). We found six genes with significant changes in isoform expression (GRMZM2G137046, GRMZM2G004483, GRMZM2G154580, GRMZM2G047055, GRMZM2G140355, and GRMZM2G021777). The GRMZM2G140355 gene resulted not statistically differentially expressed, but had significant differentially transcriptional change. Novel transcripts were discovered to be significantly expressed isoforms of two genes (GRMZ2G004483 and GRMZM2G154580) in the list (Table S2).

### qRT-PCR validation of differentially expressed transcripts

**Figure 3:**
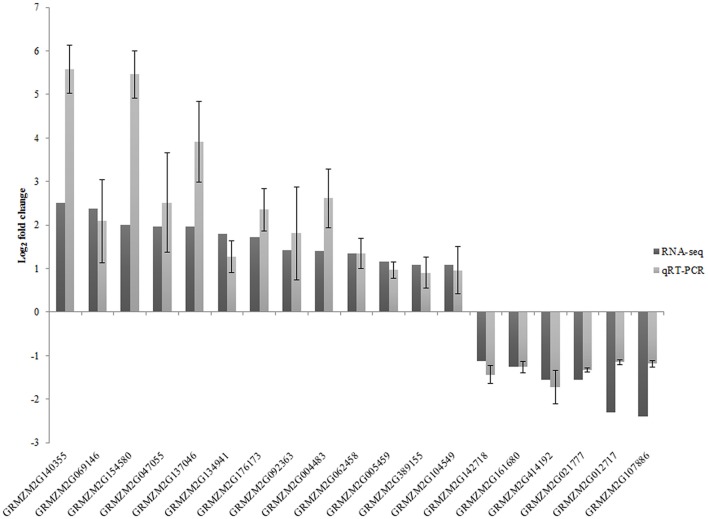
**Validation of relative gene expression obtained from RNA-seq by qRT-PCR**. Relative expression values of qRT-PCR are presented as an average *SD* of three biological replicates.

To confirm the accuracy of RNA-seq results, expression of 19 DEGs and one isoform change was analyzed by qRT-PCR for the three biological replicates (Figure 3). Except for one gene (GRMZM2G140355), which has only one isoform with significantly different expression, all of the genes were only identified as DEGs. The correlation between RNA-seq data and qRT-PCR was evaluated by comparing fold changes in gene expression. The qRT-PCR results revealed that the expression pattern of these genes was similar to that observed in the RNA-seq analysis.

To confirm alternative splicing results, the six drought-responsive isoforms (GRMZM2G137046, GRMZM2G004483, GRMZM2G154580, GRMZM2G047055, GRMZM2G140355, and GRMZM2G021777) were validated using qRT-PCR. Primers were designed to amplify a specific region of difference between isoforms, and the results of this analysis revealed that expression of the isoform was drought-responsive (Figures S2–S7), confirming RNA-seq results.

## Discussion

Various environmental stresses, such as drought, can affect maize production directly or indirectly. Over the years, numerous attempts have been made to elucidate the relationship between flowering time and drought stress by analyzing QTLs, genetic variation, and polymorphisms (Bruce et al., 2002; Franks et al., 2007; Su et al., 2013; Ziyomo and Bernardo, 2013; Xu et al., 2014; Li et al., 2016). With respect to flowering time, days to anthesis and silking are key events, and drought can increase the interval between them. Increased ASI is associated with a low rate of fertilization, which has resulted in ASI being used as an indicator of tolerance to drought stress. In order to gain insight into the molecular events that regulate flowering time in response to drought stress, we used RNA-seq. In our data, RNA-seq has identified a large number of differentially expressed transcript isoforms and novel transcripts. Therefore, to gain deeper insight into the effects of drought stress on the ASI, we explored changes in expression of genes and transcripts under drought conditions.

Flowering time is very sensitive to drought conditions, but the B73 maize strain is widely known to exhibit a smaller change in ASI in response to drought (Sari-Gorla et al., 1999; Ziyomo and Bernardo, 2013). On average, the ASI has a duration of 1 day in our plants under WW conditions, which increases to 4 days under DS conditions. We find that days to silking does not change in response to drought, but that the 3–4 day shift is due to advanced anthesis under DS conditions. Plants may advance flowering time to increase survival and ensure production of offspring under drought stress (Su et al., 2013; Kooyers, 2015). In this study, we focus on changes of drought responsive genes among flowering time genes under short-day condition. As is widely known, characteristic of B73 are temperate plant and had adapted to long-day condition. Our study has two important factors in environment, such as drought and photoperiod, which can affect flowering time genes. Considering the environment condition, we analyzed with high cut-off to more accuracy and obtained DEGs and isoforms.

Flowering time has been shown to be directly related to grain yields, therefore, many studies have explored flowering time in cereal and model plants (Buckler et al., 2009; Jung and Müller, 2009; Kong et al., 2010; Endo-Higashi and Izawa, 2011; Chen et al., 2012; Hung et al., 2012; Li et al., 2016). Based on these studies, we were able to analyze expression of homologs of genes previously reported to be related to flowering time in other plants (Li et al., 2016). Previously published expression data was in agreement with expression values from our RNA-seq data (Table S4). Our results identified 19 genes with significantly changed genes reported as candidate regulators of maize flowering time. Among these 19 genes, six genes (GRMZM2G004483, GRMZM2G154580, GRMZM2G176173, GRMZM2G092363, GRMZM2G140355, and GRMZM2G021777) were confirmed to play roles in biological processes related to flowering time, such as reproduction, flower development, and post-embryonic development (Figure 2). Changes in expression of these genes may, therefore, directly lead to changes in flowering time under drought conditions.

A subset of the drought-responsive genes we identified were found to possess CCT domains [CONSTANS, CONSTANS-LIKE, and TIMING OF CHLOROPHYLL A/B BINDING1 (TOC1)]. CCT domain-containing proteins have been shown to play a role in regulation of flowering time (Xue et al., 2008; Hung et al., 2012) and are affected by drought stress (Weng et al., 2014). Two genes (GRMZM2G09236 and GRMZM2G176173) similar to CONSTANS CO8 were found to be upregulated under drought conditions. CONSTANS CO8 genes have been reported to play a similar role to that of Grain number, plant height, and heading date7 (Ghd7), which is regarded to be a regulator of heading date and yield potential in crop plants (Coelho et al., 2013; Weng et al., 2014). In contrast, genes (GRMZM2G021777 and GRMZM2G107886) related to CONSTANS CO5 and the zinc finger-containing gene CONSTANS-LIKE 16 were downregulated. CONSTANS CO5 shares similarity with the zinc finger protein CONSTANS-LIKE 3 (COL3), which was identified as an interactor of COP1 in Arabidopsis (Datta, 2006). COP1 has been shown to regulate flowering time by acting as a repressor of flowering (Reed et al., 1994). Genes related to TOC1 exhibited opposite responses. Two genes (AC208915.3_FG010 and GRMZM2G104549) were upregulated, but a third (GRMZM2G414192) was downregulated under drought conditions. These CCT domain-containing proteins have been reported to be involved in flowering and regulation of circadian rhythms via activation of SUPPRESSOR OF CONSTANS (SOC1) (Samach, 2000; Wenkel et al., 2006). Above the CCT domains, gene belonging to the Dof family (GRMZM2G142718), which has been linked to control of flowering time (Kim et al., 2006; Fornara et al., 2009; Corrales et al., 2014) was also identified as a downregulated gene.

We also observed differential isoform expression for a subset of candidate genes (GRMZM2G137046, GRMZM2G004483, GRMZM2G154580, GRMZM2G047055, GRMZM2G140355, and GRMZM2G021777). The GRMZM2G140355 gene resulted not statistically differentially expressed, but exhibited changes in isoform expression. Novel transcripts were identified for two of these genes (GRMZM2G140355 and GRMZM2G154580). The novel transcript for GRMZM2G140355 is predicted to be related to transcription factor HY5 according to BLAST results, which, as described above, has been shown to interact directly with COP1 (Reyes et al., 2004). Another novel transcript (of GRMZM2G154580) has a blast results similar to PRR37, which has been reported to act as a floral repressor of FT-like genes, which delay flowering time in sorghum (Johnson et al., 1994b).

In response to drought, alternative splicing of the genes described above enhanced expression of the functional isoform. Alternative splicing increases functional capacity of genes, and provides an opportunity for gene regulation and another function (Yan et al., 2012). As make more than one mRNA transcript from pre-mRNA transcripts. The differential splice sites has possibility that are determined by interaction of binding proteins, transcript factors, and splicing factors which guide spliceosome (Nilsen and Graveley, 2010; Wachter et al., 2012). The consensus sequences of isoforms obtained our results (Table S2) also have potential to improve their functions, reaction rates, and give new functions.

In addition to these candidate genes, we also identified genes that appear to be related to alterations in flowering time based on BLAST results. Several genes involved in flowering time contained CCT domains and were found in the list of DEGs. Among the downregulated genes, GRMZM2G012717 is related to the zinc finger-containing gene CONSTANS-LIKE 16 (described above). A GATA transcription factor (GRMZM2G039586), which has a similar role to CONSTANS, was found in the upregulated genes (Reyes, 2004). However, FLOWERING LOCUS C (FLC), a transcriptional repressor of SOC1, was not identified as a DEG. In addition to CCT domain-containing genes, other genes related to flowering time were also found to be differentially regulated. Phytochrome kinase substrate 1 (PKS1; GRMZM2G066291), which plays a role in promoting flowering (Johnson et al., 1994b; Reed et al., 1994), was upregulated. C_2_H_2_ zinc finger gene (GRMZM2G105224), which is related to genes that control flowering time (Kim et al., 2006; Fornara et al., 2009; Corrales et al., 2014), was found in the downregulated genes (Table S1). A gene (GRMZM2G171912) related to HY5 (described above) and two genes (GRMZM2G079632 and GRMZM2G159500) with NAC domains exhibited differential isoform expression. NAC-domain proteins are known to control developmental stress responses, including flowering time (Olsen et al., 2005; Yoo et al., 2007). bHLH transcription factors have been shown to control multiple relevant processes, including flowering time and abiotic stress (Ito et al., 2012; Liu et al., 2013). In our list, two genes (GRMZM2G350312 and GRMZM2G005939) were annotated as bHLH-like transcripts. Two downregulated genes (GRMZM2G105224 and GRMZM5G801627) contained C_2_H_2_ and CCCH zinc fingers. Similar zinc finger proteins have been reported play roles in repressing flowering time (Weingartner et al., 2011; Chao et al., 2014). In addition, C_2_H_2_, bHLH, and NAC domain-containing proteins have already been reported to be important for responses to various abiotic stresses (Shinozaki and Yamaguchi-Shinozaki, 2007; Marino et al., 2008; Golldack et al., 2014). It has been suggested that some of these genes involved in abiotic stress response pathways have the potential to alter the flowering times of plants.

Alternative splicing events induced by abiotic stress have been reported in plants (Li et al., 2013; Staiger and Brown, 2013; Thatcher et al., 2016). These types of changes allow plants to respond more rapidly to environmental changes and may play important roles in survival and offspring production (Sonenberg and Hinnebusch, 2009; Lackner et al., 2012). Our results identified 617 drought-responsive DEGs and 126 isoform changes related to flowering time under drought stress. Among candidate genes previously reported to be associated with flowering time, we confirmed 19 to be drought-responsive genes. In addition to these candidate genes, we also identified new genes with potential to be involved in flowering time based on BLAST results. Some transcription factors (HY5 and PRR37) that act as repressors to proteins which may play role in delay flowering time were found to be upregulated. Similarly, known repressors of flowering time (containing C_2_H_2_ and CCCH zinc finger domains) were downregulated.

In summary, we propose that changes in expression of these drought-responsive genes advance anthesis, leading to an overall increase in the ASI. Overall, our results provide a genome-wide analysis of DEGs, novel transcripts, and isoform expression changes during the reproductive stage of maize under drought stress. Further characterization of these changes in genetic regulation will be of great value for improvement of maize breeding.

## Conclusions

In the present study, we investigated changes in transcription in response to drought stress during flowering in maize under short-day condition. Using RNA-seq, we analyzed changes in gene- and isoform-specific transcription to identify genes involved flowering time under drought stress. These genes are likely to be directly or indirectly associated with changes in the ASI in maize. Further characterization of these transcriptome changes will improve our understanding of the regulation of processes associated with flowering time under drought condition.

## Author contributions

KS and HK designed and performed experiment and KS conducted bioinformatics work, and wrote the manuscript. HK, SS, and KK have conducted field experiment and sampling. JM and JK assist bioinformatics work. BL advised on experiments and data analysis. All authors discussed the results and approved the manuscript.

### Conflict of interest statement

The authors declare that the research was conducted in the absence of any commercial or financial relationships that could be construed as a potential conflict of interest.

## Supplementary material

### Availability of supporting data

The data sets supporting the results of this article are available in the NCBI Sequence Read Archive (SRA) repository, [Http://www.ncbi.nlm.nih.gov/sra?term=SRP082534].

Figure S1 **Relative expression of *ZmDREB2A* as drought stress markers**. Relative expression values of qRT-PCR are presented as an average SD of three biological replicates.

Figure S2 **qRT-PCR validation of relative expression of isoform from drought-responsive gene (GRMZM2G004483). (A)** Sashimi plot of the gene under WW, **(B)** sashimi plot of gene under DS, **(C)** blue tracks show RNA-seq read coverage and annotated genes and red tracks show validated region using qRT-PCR, **(D)** relative expression data (log_2_ fold change) from qRT-PCR under each condition. Relative expression values of qRT-PCR are presented as an average SD of three biological replicates.

Figure S3 **qRT-PCR validation of relative expression of isoform from drought-responsive gene (GRMZM2G021777). (A)** Sashimi plot of the gene under WW, **(B)** sashimi plot of gene under DS, **(C)** blue tracks show RNA-seq read coverage and annotated genes and red tracks show validated region using qRT-PCR, **(D)** relative expression data (log_2_ fold change) from qRT-PCR under each condition. Relative expression values of qRT-PCR are presented as an average SD of three biological replicates.

Figure S4 **qRT-PCR validation of relative expression of isoform from drought-responsive gene (GRMZM2G047055). (A)** Sashimi plot of the gene under WW, **(B)** sashimi plot of gene under DS, **(C)** blue tracks show RNA-seq read coverage and annotated genes and red tracks show validated region using qRT-PCR, **(D)** relative expression data (log_2_ fold change) from qRT-PCR under each condition. Relative expression values of qRT-PCR are presented as an average SD of three biological replicates.

Figure S5 **qRT-PCR validation of relative expression of isoform from drought-responsive gene (GRMZM2G137046). (A)** Sashimi plot of the gene under WW, **(B)** sashimi plot of gene under DS, **(C)** blue tracks show RNA-seq read coverage and annotated genes and red tracks show validated region using qRT-PCR, **(D)** relative expression data (log_2_ fold change) from qRT-PCR under each condition. Relative expression values of qRT-PCR are presented as an average SD of three biological replicates.

Figure S6 **qRT-PCR validation of relative expression of isoform from drought-responsive gene (GRMZM2G140355). (A)** Sashimi plot of the gene under WW, **(B)** sashimi plot of gene under DS, **(C)** blue tracks show RNA-seq read coverage and annotated genes and red tracks show validated region using qRT-PCR, **(D)** relative expression data (log_2_ fold change) from qRT-PCR under each condition. Relative expression values of qRT-PCR are presented as an average SD of three biological replicates.

Figure S7 **qRT-PCR validation of relative expression of isoform from drought-responsive gene (GRMZM2G154580). (A)** Sashimi plot of the gene under WW, **(B)** sashimi plot of gene under DS, **(C)** blue tracks show RNA-seq read coverage and annotated genes and red tracks show validated region using qRT-PCR, **(D)** relative expression data (log_2_ fold change) from qRT-PCR under each condition. Relative expression values of qRT-PCR are presented as an average SD of three biological replicates.

Table S1 **List of differentially expressed genes (DEGs) under drought**.

Table S2 **List of differentially expressed isoforms**.

Table S3 **GO Slim terms from results of BiNGO**.

Table S4 **Expression values of candidate flowering time genes from published data**.

Table S5 **List of drought-responsive genes involved in flowering time**.

## References

[B1] Andrade F. H. Vega C. Uhart S. Cirilo A. Cantarero M. Valentinuz O. (1999). Kernel number determination in maize. Crop Sci. 39, 453 10.2135/cropsci1999.0011183X0039000200026x

[B2] Banerjee A. Roychoudhury A. (2015). WRKY proteins: signaling and regulation of expression during abiotic stress responses. Sci. World J. 2015, 1–17. 10.1155/2015/807560 25879071 PMC4387944

[B3] Benjamini Y. Yekutieli D. (2001). The control of the false discovery rate in multiple testing under dependency. Ann. Stat. 1, 1165–1188. 10.2307/2674075

[B4] Bernal M. Estiarte M. Peñuelas J. (2011). Drought advances spring growth phenology of the Mediterranean shrub *Erica multiflora*. Plant Biol. 13, 252–257. 10.1111/j.1438-8677.2010.00358.x 21309971

[B5] Blum A. (1996). Crop responses to drought and the interpretation of adaptation. Plant Growth Regul. 20, 135–148. 10.1007/BF00024010

[B6] Bolaños J. Edmeades G. O. (1996). The importance of the anthesis-silking interval in breeding for drought tolerance in tropical maize. Field Crops Res. 48, 65–80.

[B7] Bruce W. B. Edmeades G. O. Barker T. C. (2002). Molecular and physiological approaches to maize improvement for drought tolerance. J. Exp. Bot. 53, 13–25. 10.1093/jxb/53.366.13 11741036

[B8] Buckler E. S. Holland J. B. Bradbury P. J. Acharya C. B. Brown P. J. Browne C.. (2009). The genetic architecture of maize flowering time. Science 325, 714–718. 10.1126/science.1174276 19661422

[B9] Byrne P. F. Bolaños J. Edmeades G. O. Eaton D. L. (1995). Gains from selection under drought versus multilocation testing in related tropical maize populations. Crop Sci. 35, 63.

[B10] Cattivelli L. Rizza F. Badeck F.-W. Mazzucotelli E. Mastrangelo A. M. Francia E. (2008). Drought tolerance improvement in crop plants: an integrated view from breeding to genomics. Field Crops Res. 105, 1–14. 10.1016/j.fcr.2007.07.004

[B11] Chao Y. Zhang T. Yang Q. Kang J. Sun Y. Gruber M. Y.. (2014). Expression of the alfalfa CCCH-type zinc finger protein gene MsZFN delays flowering time in transgenic *Arabidopsis thaliana*. Plant Sci. 215–216, 92–99. 10.1016/j.plantsci.2013.10.012 24388519

[B12] Chen C. DeClerck G. Tian F. Spooner W. McCouch S. Buckler E. (2012). PICARA, an analytical pipeline providing probabilistic inference about *a priori* candidates genes underlying genome-wide association QTL in plants. PLoS ONE 7:e46596. 10.1371/journal.pone.0046596 23144785 PMC3492367

[B13] Coelho C. P. Costa Netto A. P. Colasanti J. Chalfun-Júnior A. (2013). A proposed model for the flowering signaling pathway of sugarcane under photoperiodic control. Genet. Mol. Res. 12, 1347–1359. 10.4238/2013.April.25.6 23661458

[B14] Conesa A. Götz S. (2008). Blast2GO: a comprehensive suite for functional analysis in plant genomics. Int. J. Plant Genomics 2008, 1–12. 10.1155/2008/619832 18483572 PMC2375974

[B15] Corrales A. R. Nebauer S. G. Carrillo L. Fernandez-Nohales P. Marques J. Renau-Morata B.. (2014). Characterization of tomato cycling Dof factors reveals conserved and new functions in the control of flowering time and abiotic stress responses. J. Exp. Bot. 65, 995–1012. 10.1093/jxb/ert451 24399177

[B16] Datta S. (2006). Arabidopsis CONSTANS-LIKE3 is a positive regulator of red light signaling and root growth. Plant Cell 18, 70–84. 10.1105/tpc.105.038182 16339850 PMC1323485

[B17] Dong Z. Danilevskaya O. Abadie T. Messina C. Coles N. Cooper M. (2012). A gene regulatory network model for floral transition of the shoot apex in maize and its dynamic modeling. PLoS ONE 7:e43450. 10.1371/journal.pone.0043450 22912876 PMC3422250

[B18] Edmeades G. O. Bänziger M. Elings A. Chapman S. C. Ribaut J. M. (1997). Recent advances in breeding for drought tolerance in maize, in Applications of Systems Approaches at the Field Level, eds Kropff M. J. Teng P. S. Aggarwal P. K. Bouma J. Bouman B. A. M. Jones J. W. van Laar H. H. (Dordrecht: Springer), 63–78. 10.1007/978-94-017-0754-1_5

[B19] Endo-Higashi N. Izawa T. (2011). Flowering time genes heading date 1 and early heading date 1 together control panicle development in rice. Plant Cell Physiol. 52, 1083–1094. 10.1093/pcp/pcr059 21565907 PMC3110884

[B20] Filichkin S. A. Priest H. D. Givan S. A. Shen R. Bryant D. W. Fox S. E.. (2010). Genome-wide mapping of alternative splicing in *Arabidopsis thaliana*. Genome Res. 20, 45–58. 10.1101/gr.093302.109 19858364 PMC2798830

[B21] Fornara F. Panigrahi K. C. Gissot L. Sauerbrunn N. Rühl M. Jarillo J. A.. (2009). Arabidopsis DOF transcription factors act redundantly to reduce CONSTANS expression and are essential for a photoperiodic flowering response. Dev. Cell 17, 75–86. 10.1016/j.devcel.2009.06.015 19619493

[B22] Franks S. J. (2011). Plasticity and evolution in drought avoidance and escape in the annual plant *Brassica rapa*. New Phytol. 190, 249–257. 10.1111/j.1469-8137.2010.03603.x 21210818

[B23] Franks S. J. Sim S. Weis A. E. (2007). Rapid evolution of flowering time by an annual plant in response to a climate fluctuation. Proc. Natl. Acad. Sci. U.S.A. 104, 1278–1282. 10.1073/pnas.0608379104 17220273 PMC1783115

[B24] Frey F. P. Urbany C. Hüttel B. Reinhardt R. Stich B. (2015). Genome-wide expression profiling and phenotypic evaluation of European maize inbreds at seedling stage in response to heat stress. BMC Genomics 16:123. 10.1186/s12864-015-1282-1 25766122 PMC4347969

[B25] Garg R. Shankar R. Thakkar B. Kudapa H. Krishnamurthy L. Mantri N.. (2016). Transcriptome analyses reveal genotype- and developmental stage-specific molecular responses to drought and salinity stresses in chickpea. Sci. Rep. 6:19228. 10.1038/srep19228 26759178 PMC4725360

[B26] Golldack D. Li C. Mohan H. Probst N. (2014). Tolerance to drought and salt stress in plants: unraveling the signaling networks. Front. Plant Sci. 5:151. 10.3389/fpls.2014.00151 24795738 PMC4001066

[B27] Hall A. J. Chimenti C. Trapani N. Vilella F. de Hunau R. C. (1984). Yield in water-stressed maize genotypes: association with traits measured in seedlings and in flowering plants. Field Crops Res. 9, 41–57. 10.1016/0378-4290(84)90005-4

[B28] Hansey C. N. Vaillancourt B. Sekhon R. S. de Leon N. Kaeppler S. M. Buell C. R. (2012). Maize (*Zea mays* L.) genome diversity as revealed by RNA-sequencing. PLoS ONE 7:e33071. 10.1371/journal.pone.0033071 22438891 PMC3306378

[B29] Hu X. Liu R. Li Y. Wang W. Tai F. Xue R. (2009). Heat shock protein 70 regulates the abscisic acid-induced antioxidant response of maize to combined drought and heat stress. Plant Growth Regul. 60, 225–235. 10.1007/s10725-009-9436-2

[B30] Humbert S. Subedi S. Cohn J. Zeng B. Bi Y. M. Chen X.. (2013). Genome-wide expression profiling of maize in response to individual and combined water and nitrogen stresses. BMC Genomics 14:3. 10.1186/1471-2164-14-3 23324127 PMC3571967

[B31] Hung H. Y. Shannon L. M. Tian F. Bradbury P. J. Chen C. Flint-Garcia S. A.. (2012). ZmCCT and the genetic basis of day-length adaptation underlying the postdomestication spread of maize. Proc. Natl. Acad. Sci. U.S.A. 109, E1913–E1921. 10.1073/pnas.1203189109 22711828 PMC3396540

[B32] Ito S. Song Y. H. Josephson-Day A. R. Miller R. J. Breton G. Olmstead R. G.. (2012). FLOWERING BHLH transcriptional activators control expression of the photoperiodic flowering regulator CONSTANS in Arabidopsis. Proc. Natl. Acad. Sci. U.S.A. 109, 3582–3587. 10.1073/pnas.1118876109 22334645 PMC3295255

[B33] Jain M. (2011). Next-generation sequencing technologies for gene expression profiling in plants. Brief. Funct. Genom. 11, elr038–elr070. 10.1093/bfgp/elr038 22155524

[B34] Jiang T. Fountain J. Davis G. Kemerait R. Scully B. Lee R. D. (2012). Root morphology and gene expression analysis in response to drought stress in maize (*Zea mays*). Plant Mol. Biol. Rep. 30, 360–369. 10.1007/s11105-011-0347-9

[B35] Johnson E. Bradley M. Harberd N. P. Whitelam G. C. (1994b). Photoresponses of light-grown phyA mutants of Arabidopsis (Phytochrome A is required for the perception of daylength extensions). Plant Physiol. 105, 141–149. 10.1104/pp.105.1.141 12232194 PMC159339

[B36] Jung C. Müller A. E. (2009). Flowering time control and applications in plant breeding. Trends Plant Sci. 14, 563–573. 10.1016/j.tplants.2009.07.005 19716745

[B37] Kakumanu A. Ambavaram M. M. Klumas C. Krishnan A. Batlang U. Myers E.. (2012). Effects of drought on gene expression in maize reproductive and leaf meristem tissue revealed by RNA-seq. Plant Physiol. 160, 846–867. 10.1104/pp.112.200444 22837360 PMC3461560

[B38] Kim D. Pertea G. Trapnell C. Pimentel H. Kelley R. Salzberg S. L. (2013). TopHat2: accurate alignment of transcriptomes in the presence of insertions, deletions and gene fusions. Genome Biol. 14:1. 10.1186/gb-2013-14-4-r36 23618408 PMC4053844

[B39] Kim S. Choi K. Park C. Hwang H. J. Lee I. (2006). SUPPRESSOR OF FRIGIDA4, encoding a C2H2-type zinc finger protein, represses flowering by transcriptional activation of Arabidopsis FLOWERING LOCUS C. Plant Cell 18, 2985–2998. 10.1105/tpc.106.045179 17138694 PMC1693938

[B40] Kong F. Liu B. Xia Z. Sato S. Kim B. M. Watanabe S.. (2010). Two coordinately regulated homologs of FLOWERING LOCUS T are involved in the control of photoperiodic flowering in soybean. Plant Physiol. 154, 1220–1231. 10.1104/pp.110.160796 20864544 PMC2971601

[B41] Kooyers N. J. (2015). The evolution of drought escape and avoidance in natural herbaceous populations. Plant Sci. 234, 155–162. 10.1016/j.plantsci.2015.02.012 25804818

[B42] Krasensky J. Jonak C. (2012). Drought, salt, and temperature stress-induced metabolic rearrangements and regulatory networks. J. Exp. Bot. 63, 1593–1608. 10.1093/jxb/err460 22291134 PMC4359903

[B43] Lackner D. H. Schmidt M. W. Wu S. Wolf D. A. Bahler J. (2012). Regulation of transcriptome, translation, and proteome in response to environmental stress in fission yeast. Genome Biol. 13:R25. 10.1186/gb-2012-13-4-r25 22512868 PMC3446299

[B44] Li W. Lin W.-D. Ray P. Lan P. Schmidt W. (2013). Genome-wide detection of condition-sensitive alternative splicing in Arabidopsis roots. Plant Physiol. 162, 1750–1763. 10.1104/pp.113.217778 23735510 PMC3700675

[B45] Li Y. X. Li C. Bradbury P. J. Liu X. Lu F. Romay C. M.. (2016). Identification of genetic variants associated with maize flowering time using an extremely large multi-genetic background population. Plant J. 86, 391–402. 10.1111/tpj.13174 27012534

[B46] Liu Y. Li X. Li K. Liu H. Lin C. (2013). Multiple bHLH proteins form heterodimers to mediate CRY2-dependent regulation of flowering-time in Arabidopsis. PLoS Genet. 9:e1003861. 10.1371/journal.pgen.1003861 24130508 PMC3794922

[B47] Liu Y. Zhou M. Gao Z. Ren W. Yang F. He H.. (2015). RNA-seq analysis reveals MAPKKK family members related to drought tolerance in maize. PLoS ONE 10:e0143128. 10.1371/journal.pone.0143128 26599013 PMC4658043

[B48] Livak K. J. Schmittgen T. D. (2001). Analysis of relative gene expression data using real-time quantitative PCR and the 2^−ΔΔCT^ method. Methods 25, 402–408. 10.1006/meth.2001.1262 11846609

[B49] Maere S. Heymans K. Kuiper M. (2005). BiNGO: a Cytoscape plugin to assess overrepresentation of gene ontology categories in biological networks. Bioinformatics 21, 3448–3449. 10.1093/bioinformatics/bti551 15972284

[B50] Marino R. Ponnaiah M. Krajewski P. Frova C. Gianfranceschi L. Pè M. E.. (2008). Addressing drought tolerance in maize by transcriptional profiling and mapping. Mol. Genet. Genomics 281, 163–179. 10.1007/s00438-008-0401-y 19018570

[B51] McMullen M. D. Kresovich S. Villeda H. S. Bradbury P. Li H. Sun Q.. (2009). Genetic properties of the maize nested association mapping population. Science 325, 737–740. 10.1126/science.1174320 19661427

[B52] Meng X. Muszynski M. G. Danilevskaya O. N. (2011). The FT-like ZCN8 gene functions as a floral activator and is involved in photoperiod sensitivity in maize. Plant Cell 23, 942–960. 10.1105/tpc.110.081406 21441432 PMC3082274

[B53] Nilsen T. W. Graveley B. R. (2010). Expansion of the eukaryotic proteome by alternative splicing. Nature 463, 457–463. 10.1038/nature08909 20110989 PMC3443858

[B54] Olsen A. N. Ernst H. A. Leggio L. L. Skriver K. (2005). NAC transcription factors: structurally distinct, functionally diverse. Trends Plant Sci. 10, 79–87. 10.1016/j.tplants.2004.12.010 15708345

[B55] Poroyko V. Spollen W. G. Hejlek L. G. Hernandez A. G. LeNoble M. E. Davis G.. (2007). Comparing regional transcript profiles from maize primary roots under well-watered and low water potential conditions. J. Exp. Bot. 58, 279–289. 10.1093/jxb/erl119 16990373

[B56] Prasch C. M. Ott K. V. Bauer H. Ache P. Hedrich R. Sonnewald U. (2015). ß-amylase1 mutant Arabidopsisplants show improved drought tolerance due to reduced starch breakdown in guard cells. J. Exp. Bot. 66, 6059–6067. 10.1093/jxb/erv323 26139825 PMC4566991

[B57] Reed J. W. Nagatani A. Elich T. D. Fagan M. Chory J. (1994). Phytochrome A and phytochrome B have overlapping but distinct functions in Arabidopsis development. Plant Physiol. 104, 1139–1149. 10.1104/pp.104.4.1139 12232154 PMC159274

[B58] Reyes J. C. (2004). The GATA family of transcription factors in Arabidopsis and rice. Plant Physiol. 134, 1718–1732. 10.1104/pp.103.037788 15084732 PMC419845

[B59] Reyes J. C. Muro-Pastor M. I. Florencio F. J. (2004). The GATA family of transcription factors in Arabidopsis and rice. Plant Physiol. 134, 1718–1732. 10.1104/pp.103.037788 15084732 PMC419845

[B60] Samach A. (2000). Distinct roles of CONSTANS target genes in reproductive development of Arabidopsis. Science 288, 1613–1616. 10.1126/science.288.5471.1613 10834834

[B61] Sari-Gorla M. Krajewski P. Di Fonzo N. Villa M. Frova C. (1999). Genetic analysis of drought tolerance in maize by molecular markers. II. Plant height and flowering. Theor. Appl. Genet. 99, 289–295. 10.1007/s001220051234

[B62] Schnable P. S. Ware D. Fulton R. S. Stein J. C. Wei F. Pasternak S.. (2009). The B73 maize genome: complexity, diversity, and dynamics. Science 326, 1112–1115. 10.1126/science.1178534 19965430

[B63] Setter T. L. Yan J. Warburton M. Ribaut J. M. Xu Y. Sawkins M.. (2010). Genetic association mapping identifies single nucleotide polymorphisms in genes that affect abscisic acid levels in maize floral tissues during drought. J. Exp. Bot. 62, 701–716. 10.1093/jxb/erq308 21084430 PMC3003815

[B64] Shankar R. Bhattacharjee A. Jain M. (2016). Transcriptome analysis in different rice cultivars provides novel insights into desiccation and salinity stress responses. Sci. Rep. 6:23719. 10.1038/srep23719 27029818 PMC4814823

[B65] Shinozaki K. Yamaguchi-Shinozaki K. (2007). Gene networks involved in drought stress response and tolerance. J. Exp. Bot. 58, 221–227. 10.1093/jxb/erl164 17075077

[B66] Sonenberg N. Hinnebusch A. G. (2009). Regulation of translation initiation in eukaryotes: mechanisms and biological targets. Cell 136, 731–745. 10.1016/j.cell.2009.01.042 19239892 PMC3610329

[B67] Staiger D. Brown J. W. (2013). Alternative splicing at the intersection of biological timing, development, and stress responses. Plant Cell 25, 3640–3656. 10.1105/tpc.113.113803 24179132 PMC3877812

[B68] Su Z. Ma X. Guo H. Sukiran N. L. Guo B. Assmann S. M.. (2013). Flower development under drought stress: morphological and transcriptomic analyses reveal acute responses and long-term acclimation in Arabidopsis. Plant Cell 25, 3785–3807. 10.1105/tpc.113.115428 24179129 PMC3877795

[B69] Thatcher S. R. Danilevskaya O. N. Meng X. Beatty M. Zastrow-Hayes G. Harris C.. (2016). Genome-wide analysis of alternative splicing during development and drought stress in maize. Plant Physiol. 170, 586–599. 10.1104/pp.15.01267 26582726 PMC4704579

[B70] Thirunavukkarasu N. Hossain F. Arora K. Sharma R. Shiriga K. Mittal S.. (2014). Functional mechanisms of drought tolerance in subtropical maize (*Zea mays* L.) identified using genome-wide association mapping. BMC Genomics 15:1182. 10.1186/1471-2164-15-1182 25539911 PMC4367829

[B71] Trapnell C. Hendrickson D. G. Sauvageau M. Goff L. Rinn J. L. Pachter L. (2012). Differential analysis of gene regulation at transcript resolution with RNA-seq. Nat. Biotechnol. 31, 46–53. 10.1038/nbt.2450 23222703 PMC3869392

[B72] Urano K. Maruyama K. Ogata Y. Morishita Y. Takeda M. Sakurai N.. (2009). Characterization of the ABA-regulated global responses to dehydration in Arabidopsis by metabolomics. Plant J. 57, 1065–1078. 10.1111/j.1365-313X.2008.03748.x 19036030

[B73] Vitting-Seerup K. Porse B. T. Sandelin A. Waage J. (2014). spliceR: an R package for classification of alternative splicing and prediction of coding potential from RNA-seq data. BMC Bioinform. 15:81. 10.1186/1471-2105-15-81 24655717 PMC3998036

[B74] Wachter A. Rühl C. Stauffer E. (2012). The role of polypyrimidine tract-binding proteins and other hnRNP proteins in plant splicing regulation. Front. Plant Sci. 3:81. 10.3389/fpls.2012.00081 22639666 PMC3355609

[B75] Wang Z. Gerstein M. Snyder M. (2009). RNA-Seq: a revolutionary tool for transcriptomics. Nat. Rev. Genet. 10, 57–63. 10.1038/nrg2484 19015660 PMC2949280

[B76] Weingartner M. Subert C. Sauer N. (2011). LATE, a C2H2 zinc-finger protein that acts as floral repressor. Plant J. 68, 681–692. 10.1111/j.1365-313X.2011.04717.x 21771123

[B77] Weng X. Wang L. Wang J. Hu Y. Du H. Xu C.. (2014). Grain number, plant height, and heading date7 is a central regulator of growth, development, and stress response. Plant Physiol. 164, 735–747. 10.1104/pp.113.231308 24390391 PMC3912102

[B78] Wenkel S. Turck F. Singer K. Gissot L. Le Gourrierec J. Samach A.. (2006). CONSTANS and the CCAAT box binding complex share a functionally important domain and interact to regulate flowering of Arabidopsis. Plant Cell Online 18, 2971–2984. 10.1105/tpc.106.043299 17138697 PMC1693937

[B79] Xu J. Yuan Y. Xu Y. Zhang G. Guo X. Wu F.. (2014). Identification of candidate genes for drought tolerance by whole-genome resequencing in maize. BMC Plant Biol. 14:83. 10.1186/1471-2229-14-83 24684805 PMC4021222

[B80] Xue W. Xing Y. Weng X. Zhao Y. Tang W. Wang L.. (2008). Natural variation in Ghd7 is an important regulator of heading date and yield potential in rice. Nat. Genet. 40, 761–767. 10.1038/ng.143 18454147

[B81] Yan K. Liu P. Wu C. Yang G. Xu R. Guo Q.. (2012). Stress-induced alternative splicing provides a mechanism for the regulation of microRNA processing in *Arabidopsis thaliana*. Mol. Cell. 48, 521–531. 10.1016/j.molcel.2012.08.032 23063528

[B82] Yoo S. Y. Kim Y. Kim S. Y. Lee J. S. Ahn J. H. (2007). Control of flowering time and cold response by a NAC-domain protein in Arabidopsis. PLoS ONE 2:e642. 10.1371/journal.pone.0000642 17653269 PMC1920552

[B83] Yu J. Holland J. B. McMullen M. D. Buckler E. S. (2008). Genetic design and statistical power of nested association mapping in maize. Genetics 178, 539–551. 10.1534/genetics.107.074245 18202393 PMC2206100

[B84] Yue G. Zhuang Y. Li Z. Sun L. Zhang J. (2008). Differential gene expression analysis of maize leaf at heading stage in response to water-deficit stress. Biosci. Rep. 28, 125–134. 10.1042/BSR20070023 18422487

[B85] Zheng J. Zhao J. Tao Y. Wang J. Liu Y. Fu J.. (2004). Isolation and analysis of water stress induced genes in maize seedlings by subtractive PCR and cDNA macroarray. Plant Mol. Biol. 55, 807–823. 10.1007/s11103-005-1969-9 15604718

[B86] Zhuang Y. Ren G. Yue G. Li Z. Qu X. Hou G.. (2007). Effects of water-deficit stress on the transcriptomes of developing immature ear and tassel in maize. Plant Cell Rep. 26, 2137–2147. 10.1007/s00299-007-0419-3 17668218

[B87] Ziyomo C. Bernardo R. (2013). Drought tolerance in maize: indirect selection through secondary traits versus genomewide selection. Crop Sci. 53, 1269 10.2135/cropsci2012.11.0651

