# Supplementary figures and images for: Transcriptome Analysis of Flowering Time Genes under Drought Stress in Maize Leaves
Source: Front Plant Sci. 2017 Mar 1;8:267. doi: 10.3389/fpls.2017.00267 (PMC5331056; doi:10.3389/fpls.2017.00267)

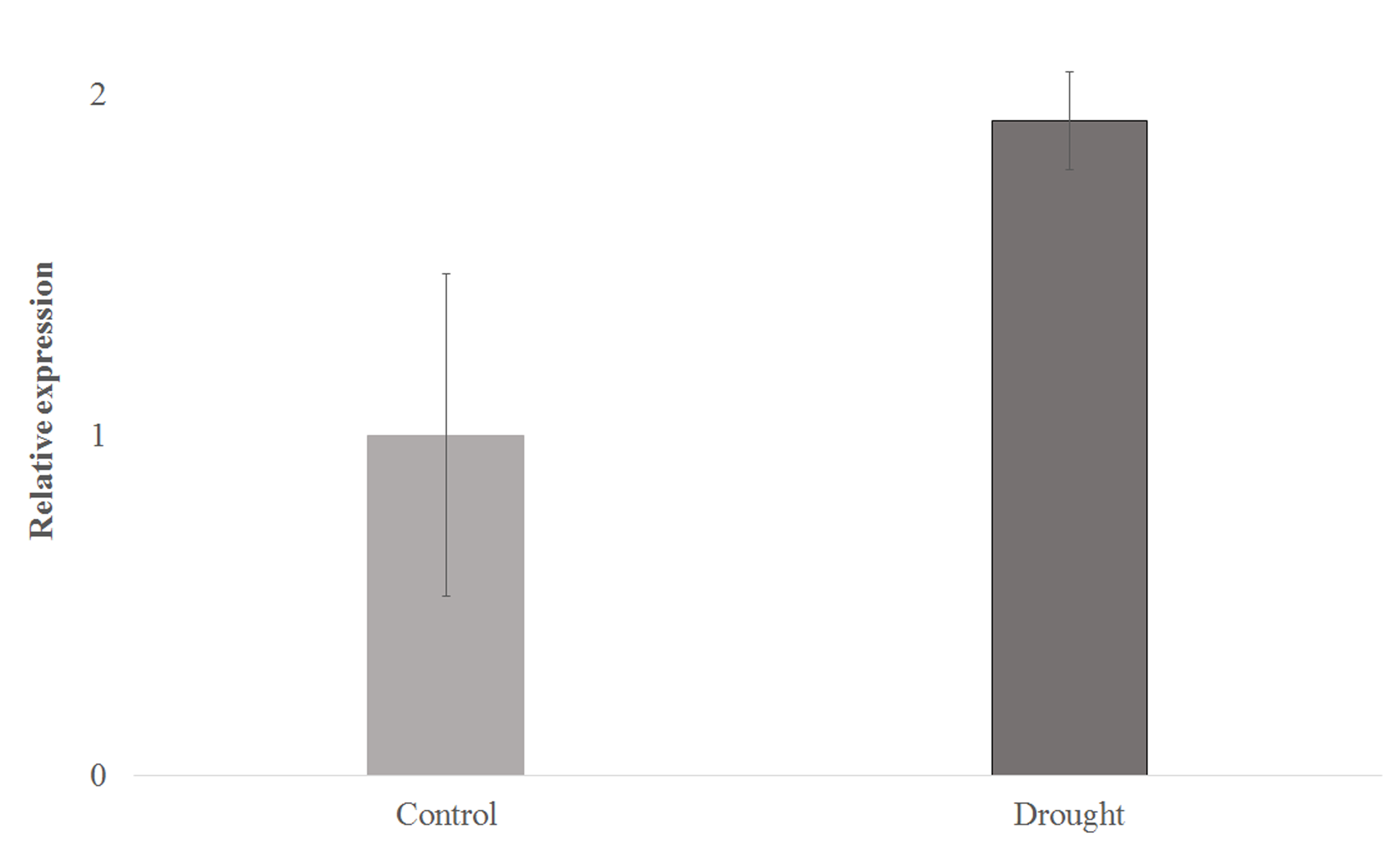

Supplement: Figure S1 — Relative expression of ZmDREB2A as drought stress markers. Relative expression values of qRT-PCR are presented as an average SD of three biological replicates. [file Image1.TIF]

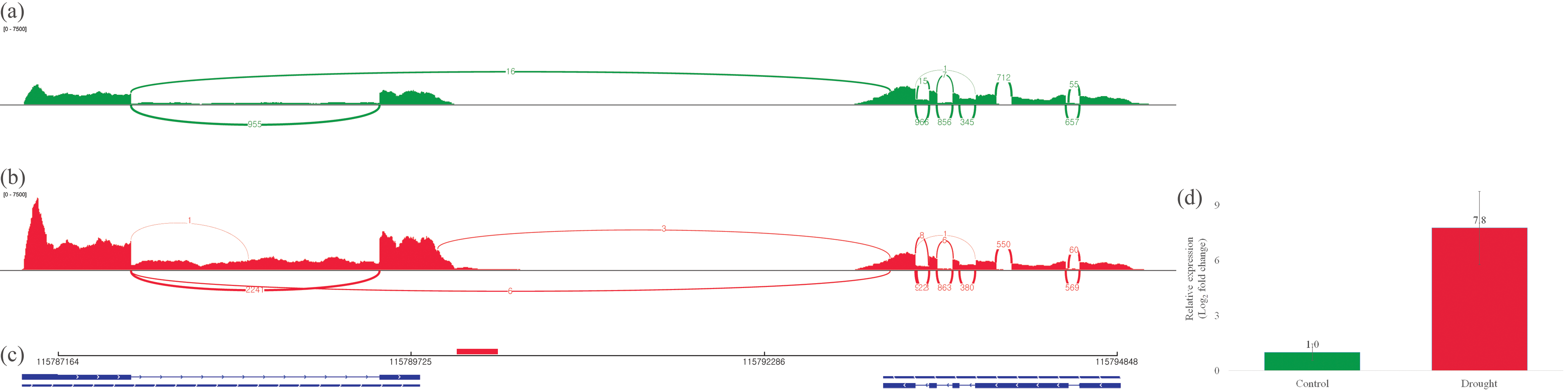

Supplement: Figure S2 — qRT-PCR validation of relative expression of isoform from drought-responsive gene (GRMZM2G004483). (A) Sashimi plot of the gene under WW, (B) sashimi plot of gene under DS, (C) blue tracks show RNA-seq read coverage and annotated genes and red tracks show validated region using qRT-PCR, (D) relative expression data (log2 fold change) from qRT-PCR under each condition. Relative expression values of qRT-PCR are presented as an average SD of three biological replicates. [file Image2.TIF]

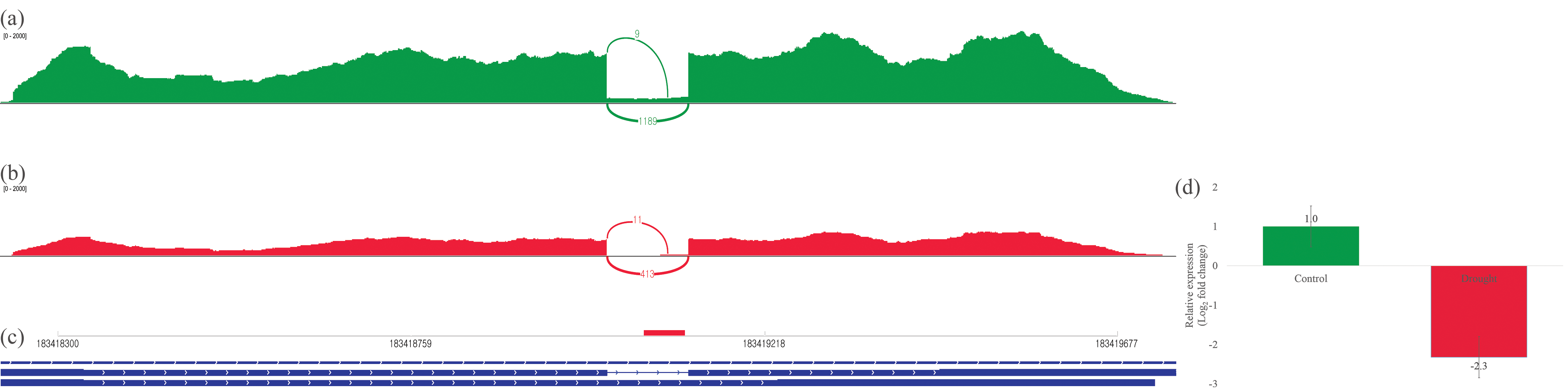

Supplement: Figure S3 — qRT-PCR validation of relative expression of isoform from drought-responsive gene (GRMZM2G021777). (A) Sashimi plot of the gene under WW, (B) sashimi plot of gene under DS, (C) blue tracks show RNA-seq read coverage and annotated genes and red tracks show validated region using qRT-PCR, (D) relative expression data (log2 fold change) from qRT-PCR under each condition. Relative expression values of qRT-PCR are presented as an average SD of three biological replicates. [file Image3.TIF]

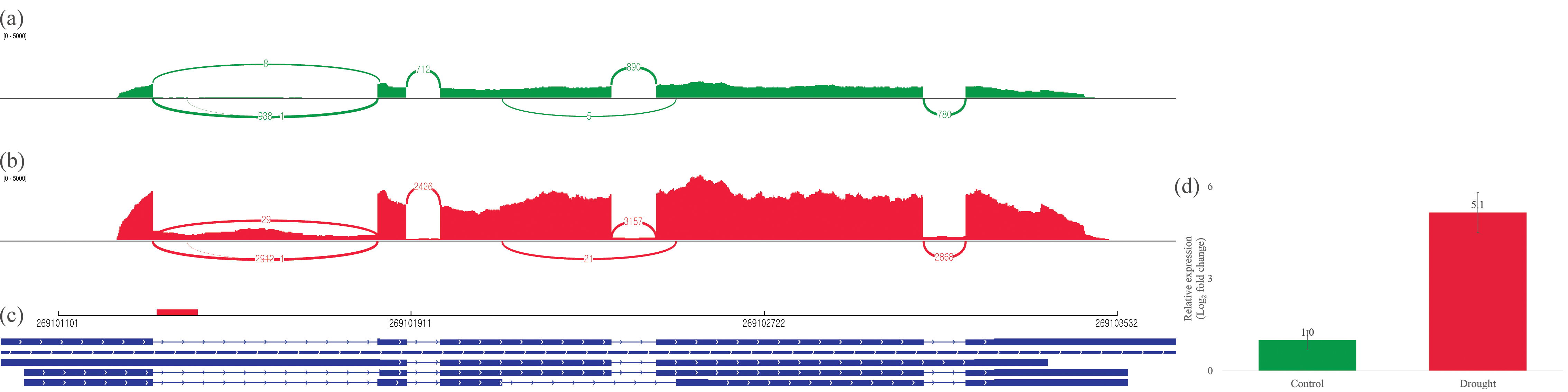

Supplement: Figure S4 — qRT-PCR validation of relative expression of isoform from drought-responsive gene (GRMZM2G047055). (A) Sashimi plot of the gene under WW, (B) sashimi plot of gene under DS, (C) blue tracks show RNA-seq read coverage and annotated genes and red tracks show validated region using qRT-PCR, (D) relative expression data (log2 fold change) from qRT-PCR under each condition. Relative expression values of qRT-PCR are presented as an average SD of three biological replicates. [file Image4.TIF]

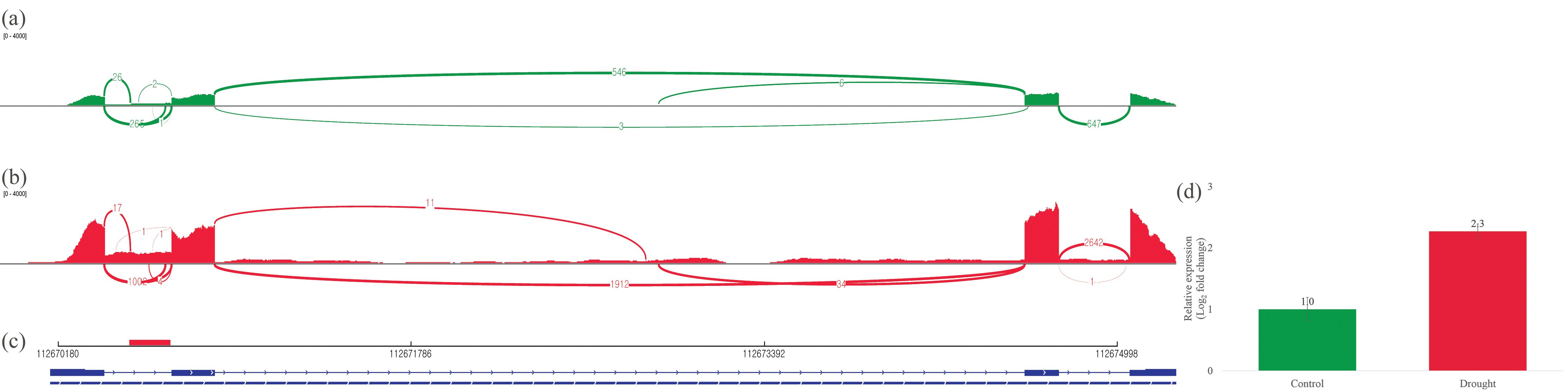

Supplement: Figure S5 — qRT-PCR validation of relative expression of isoform from drought-responsive gene (GRMZM2G137046). (A) Sashimi plot of the gene under WW, (B) sashimi plot of gene under DS, (C) blue tracks show RNA-seq read coverage and annotated genes and red tracks show validated region using qRT-PCR, (D) relative expression data (log2 fold change) from qRT-PCR under each condition. Relative expression values of qRT-PCR are presented as an average SD of three biological replicates. [file Image5.TIF]

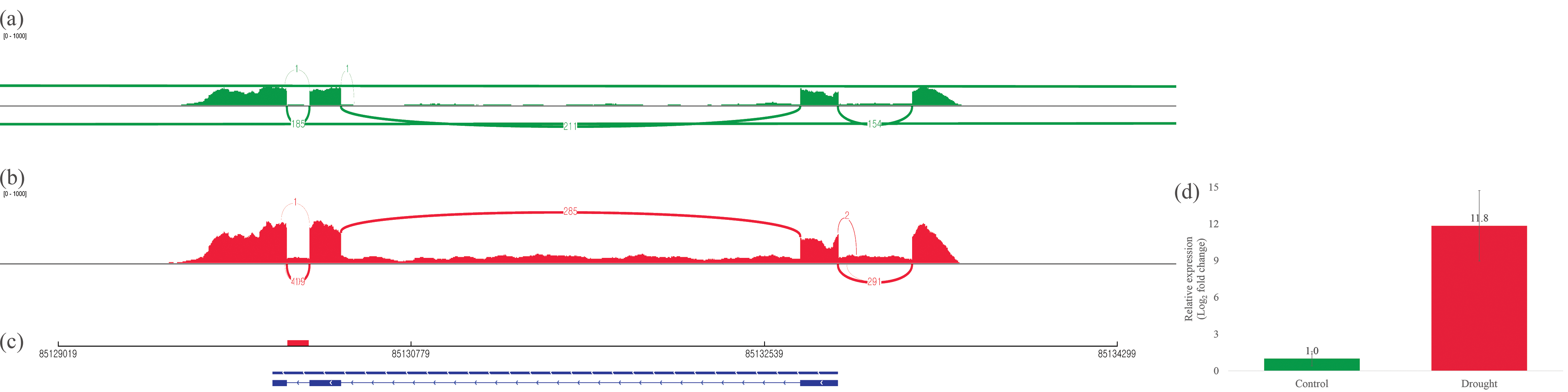

Supplement: Figure S6 — qRT-PCR validation of relative expression of isoform from drought-responsive gene (GRMZM2G140355). (A) Sashimi plot of the gene under WW, (B) sashimi plot of gene under DS, (C) blue tracks show RNA-seq read coverage and annotated genes and red tracks show validated region using qRT-PCR, (D) relative expression data (log2 fold change) from qRT-PCR under each condition. Relative expression values of qRT-PCR are presented as an average SD of three biological replicates. [file Image6.TIF]

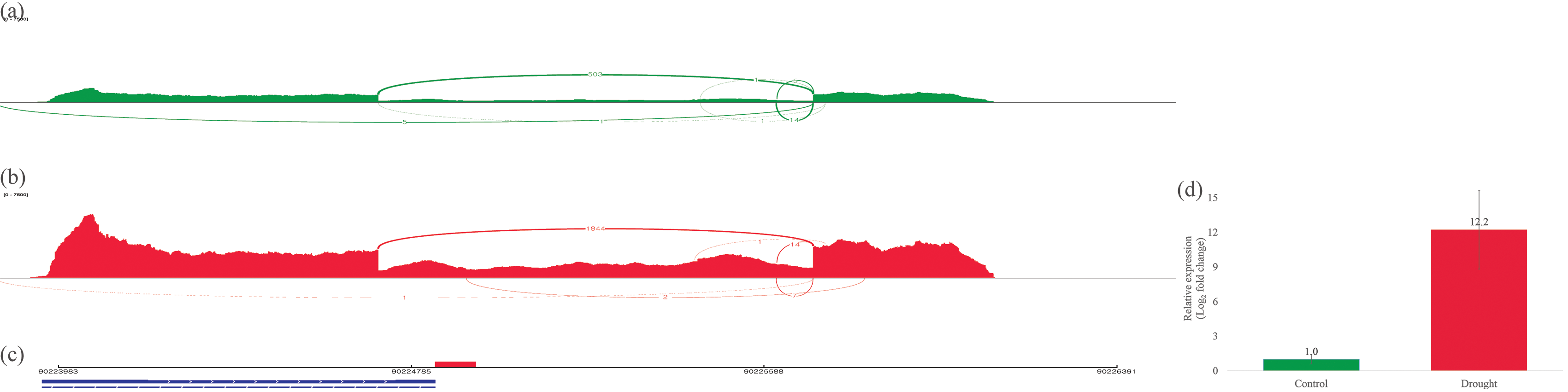

Supplement: Figure S7 — qRT-PCR validation of relative expression of isoform from drought-responsive gene (GRMZM2G154580). (A) Sashimi plot of the gene under WW, (B) sashimi plot of gene under DS, (C) blue tracks show RNA-seq read coverage and annotated genes and red tracks show validated region using qRT-PCR, (D) relative expression data (log2 fold change) from qRT-PCR under each condition. Relative expression values of qRT-PCR are presented as an average SD of three biological replicates. [file Image7.TIF]
